# The Morphological and Dynamic Changes of Ultrasound in the Evaluation of Effects of Oral Steroids Treatment for Patients with Carpal Tunnel Syndrome

**DOI:** 10.3390/diagnostics11081336

**Published:** 2021-07-26

**Authors:** Yun-Chain Yau, Chun-Pai Yang, Ching-Po Lin, I-Ju Tsai, Ching-Mao Chang, Cheng-Chia Yang, Po-Hsuan Shih, Yin-Yin Liao

**Affiliations:** 1 Department of Biomedical Imaging and Radiological Sciences, National Yang Ming Chiao Tung University, Taipei 112, Taiwan; rogeryy@gmail.com (Y.-C.Y.); chingpolin@gmail.com (C.-P.L.); 2 Department of Neurology, Kuang Tien General Hospital, Taichung 433, Taiwan; neuralyung@gmail.com; 3 Department of Nutrition, Hungkuang University, Taichung 433, Taiwan; 4 Institute of Neuroscience, National Yang Ming Chiao Tung University, Taipei 112, Taiwan; 5 Department of Medical Research, Kuang Tien General Hospital, Taichung 433, Taiwan; hunch0815@hotmail.com; 6 Center for Traditional Medicine, Taipei Veterans General Hospital, Taipei 112, Taiwan; magicbjp@gmail.com; 7 College of Medicine, National Yang Ming Chiao Tung University, Taipei 112, Taiwan; 8 Institute of Traditional Medicine, National Yang Ming Chiao Tung University, Taipei 112, Taiwan; littlerice57p@gmail.com; 9 Department of Healthcare Administration, Asia University, Taichung 413, Taiwan; chengchia@asia.edu.tw; 10 Department of Chinese Medicine, Cheng Hsin General Hospital, Taipei 112, Taiwan; 11 Department of Medical Imaging and Radiological Sciences, Chung Shan Medical University, Taichung 402, Taiwan

**Keywords:** carpal tunnel syndrome, steroid, ultrasound, cross-sectional area, amplitude

## Abstract

The role of oral steroids in carpal tunnel syndrome (CTS) remains elusive. This study aims to depict the ultrasound findings and conceivable mechanisms in relation to the efficacy of oral steroids for patients with CTS by measuring the morphological and motion changes in the median nerve. In this study, CTS patients were randomized to the oral steroid group (14 participants and 22 wrists) or nicergoline group (22 participants and 35 wrists) for 4 weeks. Both treatment arms were given global symptom score (GSS) measurements and completed an ultra-sound at baseline and at 2- and 4-weeks post-treatment. In the nerve conduction study (NCS), distal motor latency (DML) was used to assess the treatment response at baseline and 4 weeks post-treatment. The cross-sectional area (CSA) and amplitude (AMP) evaluated by the maximum lateral sliding displacement represented the morphological and dynamic changes in the median nerve, respectively. The results showed that AMP, CSA, GSS, and DML were significantly im-proved in the steroid group, as compared to the nicergoline group at weeks 2 and 4 (*p* < 0.05). The mean improvement in ultrasound parameters CSA (15.03% reduction) and AMP (466.09% increase) was better than the DML (7.88% reduction) parameter of NCS, and ultrasound changes were detectable as early as 2 weeks after oral steroid administration. Ultrasounds can serve as a tool for the quantitative measurement of treatment effects and can potentially elucidate the pathogenesis of CTS in a non-invasive and more effective manner.

## 1. Introduction

Carpal tunnel syndrome (CTS) is the most common entrapment neuropathy caused by compression of the median nerve in the wrist, with an estimated prevalence of 1–5% [1,2]. Its diagnosis can be established by a combination of clinical symptoms, physical examination, and is supported by nerve conduction studies (NCS), with or without elec-tromyography. Nevertheless, NCS is an invasive and uncomfortable measurement for the patient and has been criticized as having limited diagnostic potential, with up to 16–34% of patients clinically indicating CTS as being normal [3,4]. Recently, ultrasound im-aging has been considered to be a very useful tool in the diagnosis of CTS as it provides the capability to assess anatomical aspects of carpal tunnel and motion changes of the median nerve. A number of measurements of the median nerve, such as size, vascularity, stiffness, and mobility, have been investigated extensively [5,6,7,8,9,10]. Today, ultrasound imaging is considered to be an important alternative test for the quantitative estimation of median nerve entrapment in patients with CTS.

For ultrasound imaging, most studies have focused on investigating morphological changes in the median nerve by measuring the cross-sectional area (CSA) of the median nerve, which is the most important and commonly used ultrasound parameter for CTS diagnosis [11,12,13,14,15,16,17]. There is much less agreement as to the best cut-off value for the CSA, with the recommended cut-off values varying from 9 to 14 mm^2^ [12,13,14,15,16,17]. Moreover, the median nerve is a mobile structure that stretches, compresses and translates with the movement of the upper limb [18,19]. Several studies have also examined the mobility of the median nerve and the structure of the carpal tunnel in relation to the diagnosis of CTS [20,21,22,23,24,25,26]. The results of these studies support the thesis that patients with CTS have less longitudinal and transverse sliding of the median nerve in the affected wrist than healthy individuals during finger flexion or extension. This is because the movement of the fingers causes a rise in the tensions of the flexor digitorum superficialis and flexor digitorum profundus tendons, and the median nerve then moves in the direction of the ulnar part, where a new space is created between the tendon and the transverse carpal ligament [22,25]. This movement would pose no problem for normal median nerves. However, in patients with CTS, it is limited by the narrowed volume of the carpal tunnel or by increased pressure, usually resulting in less movement. Therefore, analyzing the nerve kinematics by observing the movement of the median nerve during wrist or finger movements is key to understanding entrapment neuropathy.

There are various treatment options for CTS, including the administration of non-steroidal anti-inflammatory drugs, oral steroids, wrist splinting, steroid injections, and surgery [27,28,29]. In conservative treatment, oral steroids provide symptom relief for patients with CTS and are frequently used in clinical practice [30,31,32,33,34,35,36]. However, the degree of improvement in patients after oral steroid treatment can always only be subjectively quantified. Moreover, the mechanism of oral steroid treatment for CTS remains elusive. Ultrasound assessment before and after the administration of oral steroids may help in establishing a more comprehensive evaluation and to improve our understanding of how oral steroids work.

The purpose of this study was to depict the ultrasound findings and conceivable mechanisms of the efficacy of oral steroids in patients with CTS by measuring the morphological changes presented by CSA and motion changes presented by lateral sliding movement patterns of the median nerve during finger flexion and extension in ultrasound B-mode images.

## 2. Materials and Methods

### 2.1. Data Acquisition

Our study protocol was approved by the Institutional Review Board of Kuang Tien General Hospital, and the recruitment period was 1 year. Several electrodiagnostic parameter estimates have been applied to the diagnosis of CTS, and this study was conducted using the standard clinical confirmatory protocol recommended by the American Association of Electrodiagnostic Medicine [37]. All patients included and analyzed in this study were clinically diagnosed with CTS, and were considered to have median neuropathy of the wrist, with the presence of at least one of the following electrodiagnostic results: (1) slow wrist to palm (antidromic) sensory nerve conduction velocity, measured velocity at a fixed distance of 8 cm less than 40 m/s; (2) slow wrist to index finger (antidromic) distal sensory latency (DSL), measured latency at a fixed distance of 14 cm proximal to the active electrode that was equal or greater than 2.5 ms; and (3) slow wrist to abductor pollicis brevis (APB) motoring, measured distal motor latency (DML) at a fixed distance of 8 cm proximal to the active electrode that was equal or greater than 4.5 ms. The severity of CTS was determined according to the rules used in previous studies, including our own [31,33,35,36]. Mild CTS refers to patients whose median sensory nerve action potential (SNAP) amplitude and compound muscle action potential (CMAP) amplitude of the APB in the palm–wrist segment remain normal, but whose conduction velocity shows a decrease and DSL was delayed. Moderate CTS refers to a condition in which the patient’s DML and DSL in the palm–wrist segment are not only abnormally delayed, but the median SNAP amplitude or CMAP amplitude of the APB muscle also decreased. Patients with CTS who had either fibrillation potentials or re-innervation on the needle electromyography of APB were considered to be severe. For patients with severe CTS, open or endoscopic surgery is generally recommended, as conservative treatment is less effective in relieving their symptoms [27,28,29,30,31]. Therefore, severe CTS was excluded from this experiment. Previous studies have shown that motor fibers might recover faster than sensory fibers after median nerve decompression; thus, most of these studies demonstrated an improvement in DML after treatment [19,33,35]. Therefore, among all NCS parameters, we selected DML in order to evaluate the treatment response.

Our study cohort included 57 wrists of 36 patients with CTS, of whom 21 had bilateral disease and 15 had unilateral disease. In order to investigate the issue of spontaneous improvement and a possible placebo effect, this study was designed to be random, double blind, and was an active controlled trial. The enrolled patients were randomly divided into 2 treatment arms: the steroid group was given 20 mg prednisolone daily for 14 days, followed by 10 mg once daily for another 14 days, and the nicergoline group was given 20 mg nicergoline daily for 14 days, followed by 10 mg once daily for another 14 days [31,33,35,36]. Computer-generated random numbers based on a stochastic indicator method were used to assign patients to different treatment groups.

Patients in both treatment arms received global symptom score (GSS) measurements and completed an ultrasound starting at the baseline, 2 weeks after, and 4 weeks after treatment. The GSS is often used in the assessment of CTS due to its moderate number of rating levels [31,32,33,35,36]. Basically, patients were asked directly, and responded subjectively, about the extent of the following five symptoms: pain, tingling, numbness, weakness/clumsiness, and nocturnal awakenings. Each symptom was rated on a scale from 0 (no symptoms) to 10 (very severe symptoms). Since the maximum score for each symptom was 10, the maximum total score for assessing the five symptoms was 50 (most severe symptoms) and the lowest score was 0 (no symptoms). Since only one hand for each individual was used in the analysis, as we included only the affected hand with the higher GSS in each individual, the number of participants was equal to the number of affected arms in the analysis group. The procedures of NCS were carried out by the same evaluator who did not have access to patient data throughout the experiment at baseline and four weeks later.

### 2.2. Ultrasound Imaging

**Figure 1:**
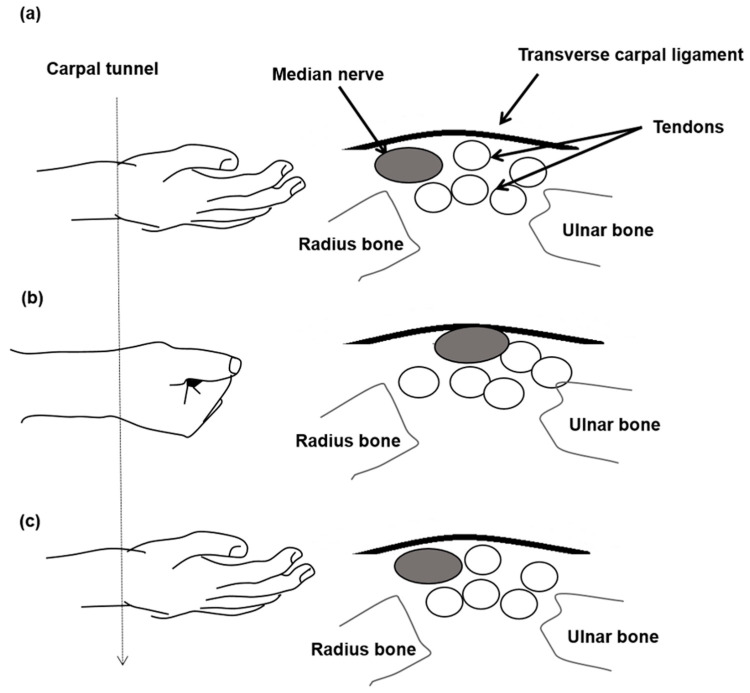
A scanned median nerve movement cycle consisted of a series of finger movements from (**a**) neutral posture to (**b**) fist clenching (finger flexion, acquisition time 1 s) and then to (**c**) palm opening (finger extension and acquisition time 2 s).

A commercial ultrasound scanner (Model t3000, Terason, Burlington, MA, USA) with a frame rate of 25 fps and a linear array probe at 10 MHz (Model 12L5A, Terason, Burlington, MA, USA) was used. The ultrasound transverse scans at the wrist level were carried out by a musculoskeletal physician who had more than 10 years of related experience. The arms of the patients were in a supine resting position when their wrists were scanned by the scanner with exactly the same settings. The transducer was placed horizontally at the level of the distal wrist crease at the entrance of the carpal tunnel and was perpendicular to the long axis of the forearm. The image depth was set to 2.5 cm. The scanning duration for obtaining the ultrasound frames along the transverse wrist line was about 2 seconds. It started with the fingers in their natural positions in the beginning (Figure 1a), and was followed by one cycle of the finger flexion–extension movement, in which the median nerve was moved towards the ulna bone with the patients fingers in a flexion first (Figure 1b), and then the radius bone with fingers in extension last (Figure 1c).

### 2.3. Median Nerve Mobility Pattern Estimation

A speckle-tracking algorithm for dynamic sequential ultrasound image scanning was applied to measure the lateral displacement of the median nerve in the radial-ulnar plane within the carpal tunnel [38]. All scanned image sequences were converted to a digital format for offline analysis on a frame-by-frame basis. The multilevel block-sum pyramid (BSP) algorithm, with a matching process and a searching process at its core, was an integrated algorithm of multilevel block-matching and BSP, and this was used for speckle tracking between sequential transverse B-mode ultrasound images in this study. This was because it had been proven to be an algorithm with excellent computational ability for two-dimensional speckle-tracking in B-mode ultrasound images. The matching process of this algorithm was based on the BSP algorithm, which not only reduced the complexity of the computation, but also maintained the same accuracy as the conventional sum of the absolute differences approach. Meanwhile, the searching process further utilized a multilevel strategy by “comparing matched blocks on a level-by-level basis” instead of using a “comprehensive search strategy”, as is the case in most conventional search methods. As the BSP enabled the elimination of unnecessary searches and the enhanced algorithm was performed in a multilevel manner from the top level down to the bottom level, the implementation of motion estimation in ultrasound images is in real-time or is in near-real-time. 

A matching block of a 32 by 32 pixels (15.56 pixels/mm) squared region in the original image, which served as the reference image, was selected for comparison with the test blocks of the same size in the search area of the target image. In order to search in finer detail, but to provide acceptable speckle-tracking results in the same time, the search block was set to a 21 by 21 pixels squared region, which was smaller than the matching block, but was large enough to cover at least 10 independent speckles. The algorithm used a loop process to compute the displacements between the test block and the matching block as the position difference of each best-matched pair of pixels between the test block (in the ith frame) of the target image and the matching block in the fourth previous image was calculated, and it continued until the displacements of all central pixels of the matching block in the reference image were obtained.

The median nerve region was the focus of this study, and we used a multilevel BSP algorithm to calculate the lateral displacement of each image pixel within the boundary of the median nerve and between the sequential cross-sectional ultrasound images. As defined in this study, the positive displacement of the median nerve corresponded to its transverse sliding motion during finger flexion toward the ulnar direction, whereas the negative displacement was toward the radial direction during finger extension. This definition was also based on the theory that the transverse movement of the median nerve over the ulnar radial axis covers the same traversal distance, and only the directions of finger flexion and extension are reversed when the patient performs a standardized repetitive finger movement. In the calculation of the median nerve displacement, all the pixels contained within its boundaries were included in the calculation. Subsequently, the average transverse displacements of the B-mode images collected at each time point during the full finger flexion and extension cycle were accumulated to obtain the cumulative lateral displacement.

One of our previous studies has demonstrated that: (i) the relationship between cumulative lateral displacement and acquisition time during a cycle of finger movements would be symmetrical with respect to the transverse sliding of the median nerve; (ii) the temporal cumulative lateral displacement could be fitted to the curve by a second-order polynomial function; and (iii) the fitted curve could be considered as the transverse sliding pattern of the median nerve during flexion and extension movements in the carpal tunnel [38]. Therefore, the amplitude (AMP) estimates represented the maximum value of the fit, indicating the maximal transverse sliding displacement of the median nerve or the spatial pressure in the carpal tunnel. To assess the morphological changes in the median nerve, the physician outlined the inner margin of the hyperechoic epineurium to measure the CSA of the median nerve with the finger in a neutral position.

### 2.4. Statistical Analysis

The sample size calculation referenced one of our previous studies that investigated an improvement in GSS from the baseline to 1 month after in the oral steroid-treated group [36]. This study was set to achieve a statistical power of 0.8 and a significance level of 0.05. Therefore, a minimum sample size of nine pairs for paired *t*-tests was required.

Differences in the distribution of age, AMP, CSA, GSS, and DML between the steroid and nicergoline groups were determined by Student’s *t*-test and were presented as mean ± standard deviation (SD). The categorical variables, including gender, side of hand, and severity, were examined by Fisher’s exact test and were expressed as a number (%). The AMP, CSA, GSS, DML were considered to be repeated measurements. The AMP, CSA, and GSS values were recorded at baseline and at 2 weeks and 4 weeks after treatment. The DML values were only recorded at baseline and 4 weeks after treatment. The least square means, mean change over time, and the difference in mean change over time between the steroid and nicergoline groups were derived from the multivariable linear mixed-effects model. The difference in mean change over time was calculated by the interaction term of time*treatment in the model. The model was adjusted for age, gender, and the side of hand. Restricted maximum likelihood estimation was used, along with unstructured covariance structure. All statistical analyses were performed with SAS 9.4 (SAS Institute, Cary, NC, USA). A two-sided *p* value of 0.05 was considered to be significant.

## 3. Results

In total, 13 right and 9 left wrists were evaluated in 14 patients, which constituted the steroid group. Additionally, 18 right and 17 left wrists were evaluated in 22 patients, which constituted the nicergoline group. There were no significant differences before treatment in gender, age, side of hands, severity, AMP, CSA, GSS, and DML between the steroid and nicergoline groups (Table 1).

In the nicergoline group, adverse effects were reported by 9% of patients, and the most frequently noted adverse effects were nausea, hot flushes, mild gastric upset, and dizziness. In the steroid group, adverse effects were reported in 21.4% of patients, and the most frequently noted adverse effects were nausea and epigastralgia. No serious adverse effects were noted in either group and no patients dropped out due to intolerance of adverse effects in this study.

**Figure 2:**
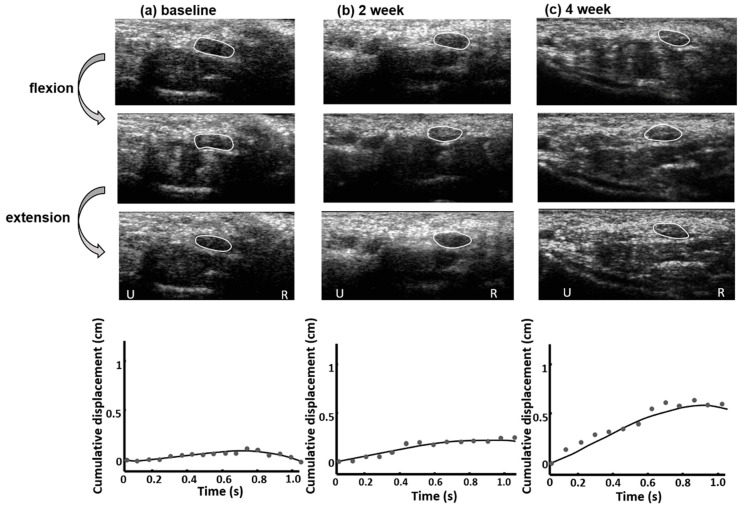
Estimation of the median nerve motion patterns in a CTS patient treated with oral steroids. At (**a**) baseline, (**b**) 2 weeks after, and (**c**) 4 weeks after treatment, the median nerve (white line) showed different transverse sliding motions over the ulnar-radial plane during finger flexion and extension movements. Cumulative displacement of the median nerve at different acquisition times during one finger flexion–extension cycle indicated the motion pattern. The dots represent the cumulative displacements at different acquisition times and the lines indicate the fitted curves for the baseline, 2 weeks after, and 4 weeks after treatment, respectively. U: ulnar side; R: radius side.

Figure 2 shows the ultrasound images of the median nerve during finger movements of a CTS patient in the steroid group. The degree and direction of transverse displacement of the median nerve varied at baseline, 2 weeks after, and 4 weeks after treatment. The transverse sliding of the median nerve during finger flexion or extension was greater at 4 weeks after treatment (Figure 2c) than at either baseline (Figure 2a) or 2 weeks after treatment (Figure 2b). After 4 weeks of oral steroid treatment, the median nerve had a larger and more regular transverse sliding motion than before treatment, and its temporally accumulated lateral displacement fitted curve showed a steady increase. Furthermore, the median nerve CSA decreased after oral steroid treatment.

**Figure 3:**
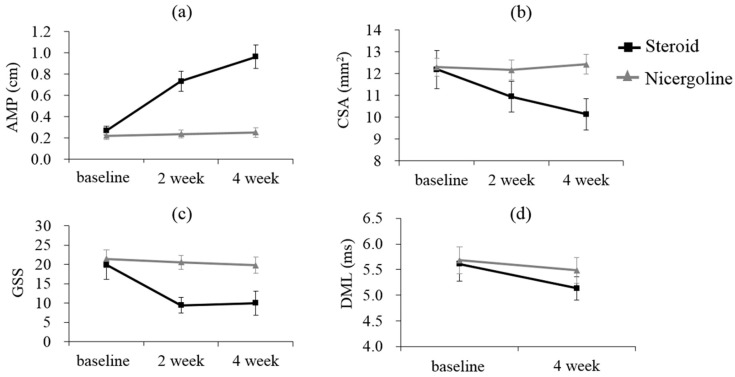
Changes in the least squares means over time in (**a**) amplitude, (**b**) cross-sectional area, (**c**) global symptom score, and (**d**) distal motor latency.

Figure 3 showed the changes over time of the AMP, CSA, GSS, and DML values of the median nerves in the steroid and nicergoline groups. Table 2 presented the least squares means of AMP, CSA, GSS, and DML values at baseline, 2 weeks after, and 4 weeks after steroid or nicergoline treatment. The AMP values increased and the CSA (except for CSA in the nicergoline group), GSS, and DML values decreased over time in both groups. After 2 or four 4 of treatment, the AMP value increased more in the steroid group than in the nicergoline group (*p* < 0.001) and the GSS value decreased much more in the steroid group than in the nicergoline group (*p* = 0.0037).

**Figure 4:**
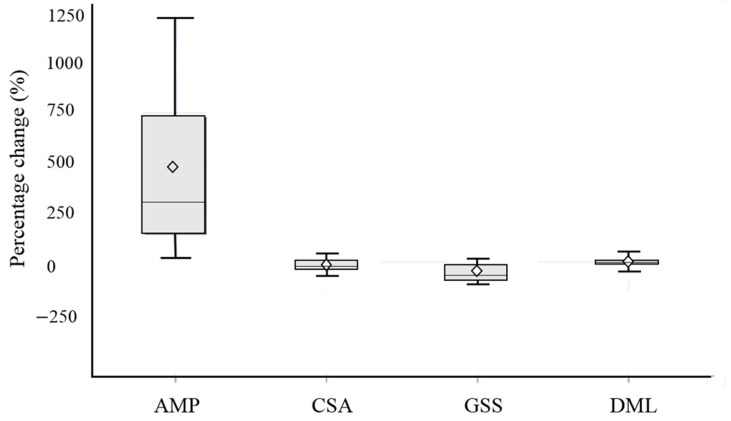
Boxplot of the percentage change in AMP, CSA, GSS, and DML values at the fourth week in the steroid group from baseline.

Moreover, the percentage changes between the baseline and the fourth week of AMP, CSA, GSS, and DML in the steroid group were shown in Figure 4. The percentage change is the ratio of the difference in quantity to its initial value multiplied by 100. The results showed an average increase of 466.09% in AMP, an average decrease of 15.03% in CSA, an average decrease of 48.86% in GSS and an average decrease of 7.88% in DML from baseline to the fourth week.

## 4. Discussion

The results of the present study show that clinical symptoms of CTS, as indicated by the GSS scores, DML values of NCS of CTS, and median nerve morphology and mobility in terms of CSA and AMP values, were significantly improved in the oral steroid group, but not in the nicergoline group. To the best of our knowledge, this is the first study to propose the use of an ultrasound model for the quantitative measurement of median nerve dysfunction after oral steroid administration, and this provides clinicians with the option of a simple, rapid, and non-invasive tool to assess treatment effects. Furthermore, it is crucial to the understanding of the plausible mechanisms of how oral steroids work and how the morphology and tissue dynamics could be observed with real-time images. The key strengths of this study include the application of a combination of validated and standardized measures of clinical assessment, NCS and ultrasound images to evaluate CTS. In addition, we adopted active drugs rather than a placebo as controls to minimize ethical issues and to encourage patients to be recruited into the current study. Moreover, all measurements were carried out at the same time and by the same group of specialists, which made it easier and more reliable to accomplish the protocol.

In the present study, the median nerve responded positively to oral steroid treatment during the first two weeks, demonstrating decreased CSA and GSS and increased AMP. It seems that ultrasound parameters could be used to detect the morphological and mobility recovery of the median nerve very early after oral steroid use. The improvements observed with an ultrasound were maintained over the subsequent 2 weeks of treatment, and persistent improvement was evident. The morphological changes, in the present study, of the median nerve over a period of 4 weeks of oral steroid administration, showed a mean decrease in CSA of 15.03%, while the movement changes showed a mean increase in AMP of 466.09%. After 4 weeks, the NCS revealed a decrease in DML values, but this improvement did not return to normal levels. As can be seen from the results, the DML values decreased by an average of 7.88% from baseline to week four, which was a lower change rate than the ultrasound measurements. The results of this study can support the notion that ultrasound parameters have an advantage over NCS in quantifying the degree of improvement in CTS patients after treatment. The above-mentioned results are yet to be further confirmed by future studies with larger sample sizes. 

While the pathophysiology of CTS is not fully understood, CTS is thought to involve inflammation and ischemic injury of the median nerve caused by elevated pressure within the carpal tunnel, which in turn leads to exudative edema, and subsequent fibrosis [39]. Management of CTS is based on disease severity. For patients with severe CTS, conservative treatment, including the administration of non-steroidal anti-inflammatory drugs, oral steroids, wrist splints, and steroid injections, which have been suggested for mild or moderate CTS, is less effective in reducing CTS symptoms [27,28,29,30,31]. Open or endoscopic surgery is recommended for patients with severe median nerve damage characterized by sustained axonal loss or denervation in electrodiagnostic studies [29,34]. Therefore, conservative treatment, such as oral steroid therapy, could be given to patients with CTS who do not fulfill surgical indication. Previous studies confirmed that short-term and low dose oral steroids (20 mg prednisolone daily for 14 days followed by 10 mg once daily for another 14 days) could be of great benefit to patients with mild or moderate CTS and serious adverse effects could be avoided [31,33,35,36]. It is generally assumed that steroids are effective in reducing swelling because of their anti-inflammatory response. However, the role and mechanism of oral steroid therapy in patients with CTS after its administration remains uncertain and unquantified. It is generally believed that the increased CSA of the median nerve in patients with CTS is due to edema and fibrotic proliferation [19,40]. Notably, our previous studies have shown that finger movements in normal subjects reveal greater lateral sliding of the median nerve in 2D dynamic ultrasound images and a steeper fitted curve when compared to patients with mild or severe CTS [38]. In this study, we used 2D dynamic ultrasound images to assess the morphology and motion of the median nerve during finger movements in patients with CTS treated with oral steroids or nicergoline. We found that only in patients treated with oral steroids was the size of the median nerve presented by CSA decreased and the transverse sliding motion of the median nerve and the curvature of its fitted curve increased; this was not the case in patients treated with nicergoline. In addition, the improvement in the steroid group cannot be explained by spontaneous improvement or merely placebo effects, because the randomization means that spontaneous and placebo effects occurred evenly in both groups. Although we did find that oral steroids were effective in the treatment of CTS, with no serious adverse effects during 4 weeks of treatment, 21.4% of patients in the steroid group experienced adverse effects, which was greater than the reported 9% of patients in the nicergoline group.

There are several limitations of this study. First, the sample size was small and the observation period was relatively short. The effects of short-term and low-dose steroids can last up to 8 weeks in patients with mild or moderate CTS [28,30,31,33,35]. Due to this, the effectiveness of ultrasounds could be observed over a period of 2 months and more significant improvements may be found at the end of the second month. However, ultrasound parameters might be affected by decreased drug efficacy or symptom relapse as the time increased. Larger and longer periods of clinical studies, including a continuous observation of ultrasound changes over time, are needed to corroborate our findings. Second, only one musculoskeletal physician performed all the studies to minimize potential measurement variability, but the consistency of the individual’s self-reliability is worth noting. Third, we only identified the morphologic and motion changes of the median nerve in patients with CTS. If the elasticity of the median nerve, which is thought to be afflicted by neuropathy and has higher values, is included in further studies, this may provide further evidence regarding the pathogenesis of CTS and its response to treatment [10,41,42]. Fourth, the body mass index and wrist circumference were not taken into consideration in patient assessment. These variables represent the possible effects in the carpal tunnel and median nerve areas, and might have contributed to further statistical bias [43,44].

## 5. Conclusions

In conclusion, the ultrasound study of the median nerve demonstrated significant changes in CSA and AMP following oral steroid treatment for CTS. Ultrasound may serve as a potentially useful follow-up tool to assess the morphological and mobility changes in CTS patients after oral steroid administration, and the results may have implications for clinicians in utilizing ultrasounds to evaluate the outcomes of interventions and to further elucidate the pathogenesis of CTS in the future.

## Figures and Tables

**Table 1:** Baseline characteristics.

Variable	Steroid (*n* = 14, 22 Wrists)	Nicergoline (*n* = 22, 35 Wrists)	*p*-Value
Age (years), mean ± SD	58.00 ± 11.29	52.95 ± 7.16	0.109
Gender, *n* (%)			
Male	5 (35.71%)	3 (13.63%)	0.217
Female	9 (64.28%)	19 (86.36%)	
Side of hands, *n* (%)			
Left	1 (7.14%)	4 (18.18)	0.528
Right	5 (35.71%)	5 (22.73%)	
Both	8 (57.14%)	13 (53.09%)	
Severity, *n* (%)			
Mild	4 (28.57%)	2 (9.09%)	0.181
Moderate	10 (71.43%)	20 (90.91%)	
AMP (cm), mean ± SD	0.25 ± 0.16	0.25 ± 0.09	0.912
CSA (mm^2^), mean ± SD	12.50 ± 3.39	12.25 ± 2.08	0.807
GSS, mean ± SD	10.86 ± 13.11	20.36 ± 9.48	0.692
DML (ms), mean ± SD	5.68 ± 1.29	5.63 ± 1.20	0.899

Notes: AMP, amplitude; CSA, cross-sectional area; GSS, global symptom score; DML, distal motor latency.

**Table 2:** Least squares means of AMP, CSA, GSS, and DML at the 1st (baseline), 2nd, and 4th week with a mixed-effects model *.

Variable	Steroid		Nicergoline		Difference in Change over Time	*p*-Value
	Mean (SE)	within Group Change	Mean (SE)	within Group Change		
AMP (cm)						
baseline	0.27 (0.04)	reference	0.22(0.04)	reference	reference	
2 week	0.73 (0.09)	0.46 (0.10)	0.24(0.04)	0.01 (0.02)	0.45 (0.10)	<0.001
4 week	0.96 (0.11)	0.69 (0.11)	0.25 (0.04)	0.03 (0.02)	0.66 (0.11)	<0.001
CSA (mm^2^)						
baseline	12.18 (0.86)	reference	12.29 (0.42)	reference	reference	
2 week	10.94 (0.69)	−1.24 (0.50)	12.16 (0.46)	−0.13 (0.34)	−1.12 (0.60)	0.0665
4 week	10.13 (0.72)	−2.05 (0.90)	12.42 (0.45)	0.13 (0.35)	−2.18 (0.96)	0.0255
GSS						
baseline	19.97 (3.86)	reference	21.47 (2.35)	reference	reference	
2 week	9.38 (2.03)	−10.6 (3.09)	20.59 (1.81)	−0.89 (1.10)	−9.71 (3.28)	0.0037
4 week	9.97 (3.09)	−10.0 (4.28)	19.79 (2.12)	−1.69 (1.43)	−8.31 (4.51)	0.0676
DML (ms)						
baseline	5.61 (0.33)	reference	5.68 (0.26)	reference	reference	
4 week	5.13 (0.23)	−0.47 (0.24)	5.48 (0.25)	−0.19 (0.12)	−0.28 (0.27)	0.297

* Adjusted for age, gender, and side of hand. Difference in change over time: mean change of the steroid group—mean change of the nicergoline group. Notes: AMP, amplitude; CSA, cross-sectional area; GSS, global symptom score; DML, distal motor latency.

## Data Availability

Data available on request due to privacy and ethical restrictions.

## References

[1] Werner R.A., Andary M. (2002). Carpal tunnel syndrome: Pathophysiology and clinical neurophysiology. Clin. Neurophysiol..

[2] Dale A.M., Harris-Adamson C., Rempel D., Gerr F., Hegmann K., Silverstein B., Burt S., Garg A., Kapellusch J., Merlino L. (2013). Prevalence and incidence of carpal tunnel syndrome in US working populations: Pooled analysis of six prospective studies. Scand. J. Work. Environ. Health.

[3] Witt J.C., Hentz J.G., Stevens J.C. (2004). Carpal tunnel syndrome with normal nerve conduction studies. Muscle Nerve.

[4] Atroshi I., Gummesson C., Johnsson R., Ornstein E. (2003). Diagnostic properties of nerve conduction tests in population-based carpal tunnel syndrome. BMC Musculoskelet. Disord..

[5] Keberle M., Jenett M., Kenn W., Reiners K., Peter M., Haerten R., Hahn D. (2000). Technical advances in ultrasound and MR imaging of carpal tunnel syndrome. Eur. Radiol..

[6] Nakamichi K.-I., Tachibana S. (2002). Ultrasonographic measurement of median nerve cross-sectional area in idiopathic carpal tunnel syndrome: Diagnostic accuracy. Muscle Nerve.

[7] Lee C.H., Kim T.K., Yoon E.S., Dhong E.S. (2005). Correlation of High-Resolution Ultrasonographic Findings with the Clinical Symptoms and Electrodiagnostic Data in Carpal Tunnel Syndrome. Ann. Plast. Surg..

[8] Yoshii Y., Villarraga H.R., Henderson J., Zhao C., An K.-N., Amadio P.C. (2009). Speckle Tracking Ultrasound for Assessment of the Relative Motion of Flexor Tendon and Subsynovial Connective Tissue in the Human Carpal Tunnel. Ultrasound Med. Biol..

[9] Akcar N., Özkan S., Mehmetoglu Ö., Calisir C., Adapinar B. (2010). Value of Power Doppler and Gray-Scale US in the Diagnosis of Carpal Tunnel Syndrome: Contribution of Cross-Sectional Area just before the Tunnel Inlet as Compared with the Cross-Sectional Area at the Tunnel. Korean J. Radiol..

[10] Kantarci F., Ustabasioglu F.E., Delil S., Olgun D.C., Korkmazer B., Dikici A.S., Tutar O., Nalbantoglu M., Uzun N., Mihmanli I. (2013). Median nerve stiffness measurement by shear wave elastography: A potential sonographic method in the diagnosis of carpal tunnel syndrome. Eur. Radiol..

[11] McDonagh C., Alexander M., Kane D. (2014). The role of ultrasound in the diagnosis and management of carpal tunnel syndrome: A new paradigm. Rheumatology.

[12] Sarría L., Cabada T., Cozcolluela R., Martínez-Berganza T., García S. (2000). Carpal tunnel syndrome: Usefulness of sonography. Eur. Radiol..

[13] Wong S.M., Griffith J., Hui A.C.F., Lo S.K., Fu M., Wong K.S.L. (2004). Carpal Tunnel Syndrome: Diagnostic Usefulness of Sonography. Radiology.

[14] Moran-Blanco L.M., Perez M., Esteban A., Bellon J., Arranz B., Del Cerro M. (2009). Sonographic measurement of cross-sectional area of the median nerve in the diagnosis of carpal tunnel syndrome: Correlation with nerve conduction studies. J. Clin. Ultrasound.

[15] Fowler J.R., Gaughan J.P., Ilyas A.M. (2011). The Sensitivity and Specificity of Ultrasound for the Diagnosis of Carpal Tunnel Syndrome: A Meta-analysis. Clin. Orthop. Relat. Res..

[16] Billakota S., Hobson-Webb L.D. (2017). Standard median nerve ultrasound in carpal tunnel syndrome: A retrospective review of 1021 cases. Clin. Neurophysiol. Pract..

[17] Yoshii Y., Zhao C., Amadio P.C. (2020). Recent Advances in Ultrasound Diagnosis of Carpal Tunnel Syndrome. Diagnostics.

[18] Yoshii Y., Villarraga H.R., Henderson J., Zhao C., An K.-N., Amadio P.C. (2009). Ultrasound Assessment of the Displacement and Deformation of the Median Nerve in the Human Carpal Tunnel with Active Finger Motion. J. Bone Jt. Surg. Am..

[19] Yao Y., Grandy E., Jenkins L., Hou J., Evans P.J., Seitz W.H., Li Z.-M. (2019). Changes of median nerve conduction, cross-sectional area and mobility by radioulnar wrist compression intervention in patients with carpal tunnel syndrome. J. Orthop. Transl..

[20] Nakamichi K., Tachibana S. (1992). Transverse Sliding of the Median Nerve Beneath the Flexor Retinaculum. J. Hand Surg..

[21] Nakamichi K., Tachibana S. (1995). Restricted Motion of the Median Nerve in Carpal Tunnel Syndrome. J. Hand Surg..

[22] Wright T.W., Glowczewskie F., Wheeler D., Miller G., Cowin D. (1996). Excursion and Strain of the Median Nerve. J. Bone Jt. Surg. Am..

[23] Erel E., Dilley A., Greening J., Morris V., Cohen B., Lynn B. (2003). Longitudinal sliding of the median nerve in patients with carpal tunnel syndrome. J. Hand Surg..

[24] Osamura N., Zhao C., Zobitz M.E., An K.-N., Amadio P.C. (2007). Evaluation of the material properties of the subsynovial connective tissue in carpal tunnel syndrome. Clin. Biomech..

[25] Liao Y.-Y., Lee W.-N., Lee M.-R., Chen W.-S., Chiou H.-J., Kuo T.-T., Yeh C.-K. (2015). Carpal Tunnel Syndrome: US Strain Imaging for Diagnosis. Radiology.

[26] Moon H., Lee B.J., Park D. (2020). Change to movement and morphology of the median nerve resulting from steroid injection in patients with mild carpal tunnel syndrome. Sci. Rep..

[27] Gerritsen A.A.M., De Krom M.C.T.F.M., Struijs M.A., Scholten R.J.P.M., De Vet H.C.W., Bouter L.M. (2000). Conservative treatment options for carpal tunnel syndrome: A systematic review of randomised controlled trials. J. Neurol..

[28] Huisstede B.M., Hoogvliet P., Randsdorp M.S., Glerum S., van Middelkoop M., Koes B. (2010). Carpal Tunnel Syndrome. Part I: Effectiveness of Nonsurgical Treatments—A Systematic Review. Arch. Phys. Med. Rehabil..

[29] Wipperman J., Goerl K. (2016). Carpal tunnel syndrome: Diagnosis and management. Am. Fam. Physician.

[30] Herskovitz S., Berger A.R., Lipton R.B. (1995). Low-dose, short-term oral prednisone in the treatment of carpal tunnel syndrome. Neurology.

[31] Chang M.H., Chiang H.T., Lee S.S.-J., Ger L.P., Lo Y.K. (1998). Oral drug of choice in carpal tunnel syndrome. Neurology.

[32] Hui A.C.F., Wong S.M., Wong K.S.L., Li E., Kay R., Yung P.S.-H., Hung L.K., Yu L.M. (2001). Oral steroid in the treatment of carpal tunnel syndrome. Ann. Rheum. Dis..

[33] Chang M.-H., Ger L.-P., Hsieh P.F., Huang S.-Y. (2002). A randomised clinical trial of oral steroids in the treatment of carpal tunnel syndrome: A long term follow up. J. Neurol. Neurosurg. Psychiatry.

[34] Viera A.J. (2003). Management of carpal tunnel syndrome. Am. Fam. Physician.

[35] Yang C.-P., Hsieh C.-L., Wang N.-H., Li T.-C., Hwang K.-L., Yu S.-C., Chang M.-H. (2009). Acupuncture in Patients with Carpal Tunnel Syndrome. Clin. J. Pain.

[36] Yang C.-P., Wang N.-H., Li T.-C., Hsieh C.-L., Chang H.-H., Hwang K.-L., Ko W.-S., Chang M.-H. (2011). A Randomized Clinical Trial of Acupuncture Versus Oral Steroids for Carpal Tunnel Syndrome: A Long-Term Follow-Up. J. Pain.

[37] Stevens J.C. (1997). AAEM minimonograph #26: The electrodiagnosis of carpal tunnel syndrome. American Association of Elec-trodiagnostic Medicine. Muscle Nerve.

[38] Kuo T.-T., Lee M.-R., Liao Y.-Y., Chen J.-P., Hsu Y.-W., Yeh C.-K. (2016). Assessment of Median Nerve Mobility by Ultrasound Dynamic Imaging for Diagnosing Carpal Tunnel Syndrome. PLoS ONE.

[39] Aboonq M.S. (2015). Pathophysiology of carpal tunnel syndrome. Neurosciences.

[40] Vögelin E., Nüesch E., Jüni P., Reichenbach S., Eser P., Ziswiler H.-R. (2010). Sonographic Follow-Up of Patients with Carpal Tunnel Syndrome Undergoing Surgical or Nonsurgical Treatment: Prospective Cohort Study. J. Hand Surg..

[41] Asadov R., Erdal A., Buğdaycı O., Gündüz O.H., Ekinci G. (2018). The effectiveness of ultrasonography and ultrasonographic elastography in the diagnosis of carpal tunnel syndrome and evaluation of treatment response after steroid injection. Eur. J. Radiol..

[42] Cingoz M., Kandemirli S.G., Alis D.C., Samanci C., Kandemirli G.C., Adatepe N.U. (2018). Evaluation of median nerve by shear wave elastography and diffusion tensor imaging in carpal tunnel syndrome. Eur. J. Radiol..

[43] Kurt S., Kisacik B., Kaplan Y., Yildirim B., Etikan I., Karaer H. (2008). Obesity and Carpal Tunnel Syndrome: Is There a Causal Relationship?. Eur. Neurol..

[44] Komurcu H.F., Kilic S., Anlar O. (2014). Relationship of Age, Body Mass Index, Wrist and Waist Circumferences to Carpal Tunnel Syndrome Severity. Neurol. Med. Chir..

